# Multiple Paragangliomas in the Carotid Body, Adrenal and Extra-Adrenal Retroperitoneal Locations

**DOI:** 10.7759/cureus.18258

**Published:** 2021-09-24

**Authors:** Tushar Kalekar, Varsha Rangankar, Dileep Reddy Ayapaneni, Vijetha Chanabasanavar, Satvik Dhirawani

**Affiliations:** 1 Department of Radiology, Dr. D. Y. Patil Medical College, Hospital and Research Centre, Pune, IND

**Keywords:** paraaortic, carotid body, extra-adrenal, multiple, pheochromocytoma

## Abstract

Paragangliomas are chromaffin cell tumors that arise from neural crest cells and are extremely rare. Multiple paragangliomas in different locations of the neck and abdomen in the same patient are highly uncommon. We give the instance of a hypertensive male aged 42 years with a history of breathlessness, chest pain, and excessive perspiration for 10 days. Computed tomography of neck and abdomen revealed solid homogenous intensely enhancing masses in the left adrenal of size 64 x 45 x 52 mm [AP x TR x CC (anteroposterior x transverse x craniocaudal)], left paraaortic region of size 41 x 28 x 29 mm [CC x TR x AP (craniocaudal x transverse x anteroposterior)] and at the division of the left common carotid artery of size 17 x 15 x 11 mm (CC x TR x AP). The patient underwent a diagnostic laparotomy and resected tumors were diagnosed as paragangliomas. The possibility of paragangliomas should always be considered when hypervascular masses are encountered in certain locations of the body. Presence of such a lesion must prompt further imaging of the common sites of paragangliomas for the detection of occult synchronous paragangliomas. Routine screening at timely intervals in patients previously diagnosed with paraganglioma may aid in the earlier detection of metachronous tumors.

## Introduction

Paraganglia are clusters of neuroendocrine cells found throughout the body in close relation to the autonomic nervous system and work in either a parasympathetic or sympathetic manner. The adrenal medulla has the biggest group of cells, followed by the paravertebral space, head, and neck region. Paragangliomas (PGL) are tumors that arise in the paraganglia. Paraganglioma accounts for 10-18% of all chromaffin tissue-related tumors [1].

Parasympathetic paragangliomas are non-secretory tumors that originate in the paraganglia of the head and neck. They are associated with mass-effect symptoms such as cranial nerve palsies, neck mass, or tinnitus. They make up roughly 0.6% of head and neck neoplasms. They occur most commonly in the carotid body, jugular body, vagus nerve, and glossopharyngeal nerve [2].

Sympathetic paragangliomas are tumors that develop in paraganglia below the level of the neck and are symmetrically distributed along the aorta, adjacent to the sympathetic chain. They either form intraadrenal paragangliomas, commonly known as pheochromocytoma, in the adrenal medulla or persists in its paraaortic locations, where it can form extra-adrenal, retroperitoneal paragangliomas. They are secretory and show signs of catecholamine excess, including headaches, palpitations, diaphoresis, and hypertension [3].

Paragangliomas can be evaluated using a variety of radiologic and nuclear imaging techniques. Magnetic resonance imaging (MRI) and computed tomography (CT) are mainly used for their diagnosis. Functional imaging modalities such as meta-iodobenzylguanidine (MIBG) scintigraphy and 18F-fluorodeoxyglucose positron emission tomography/computed tomography (18F-FDG PET/CT) help discover additional lesions that can influence the surgical approach [4, 5].

Multiple paragangliomas can present synchronously or metachronously. Multicentric tumors are seen in 10-20% of paragangliomas, which are more frequent in the head and neck region [6]. In the present case, the multiple paragangliomas were located in different locations of the head and neck and adrenal and extra-adrenal sites of retroperitoneum.

## Case presentation

A male aged 42 years, a known case of type 2 diabetes and recently diagnosed hypertension complained of acute onset breathlessness with chest pain. The patient also complained of headaches, palpitations, and excessive sweating for the past two months. No significant past medical, family and psychosocial history including relevant genetic information was present.

On examination, he had raised blood pressure of 152/92 mmHg. There was no orthostatic hypotension. The electrocardiogram showed sinus rhythm (98 beats/min) with left ventricular hypertrophy (LVH) signs. The plasma proBNP (proB-type natriuretic peptide) was in the normal range (80 pg/ml; normal values < 100 pg/ml). Other investigations i.e. electrolytes, creatinine, and troponin were within normal limits. A plain chest radiograph revealed normal lungs and cardiac silhouette. A transthoracic 2D-echocardiogram revealed severe left ventricle hypertrophy (end-diastolic intraventricular septum thickness-19 mm) with dilatation of the left atrium (22 cm^2^) and normal left ventricular ejection fraction (LVEF-62%) suggestive of hypertrophic obstructive cardiomyopathy.

**Figure 1:**
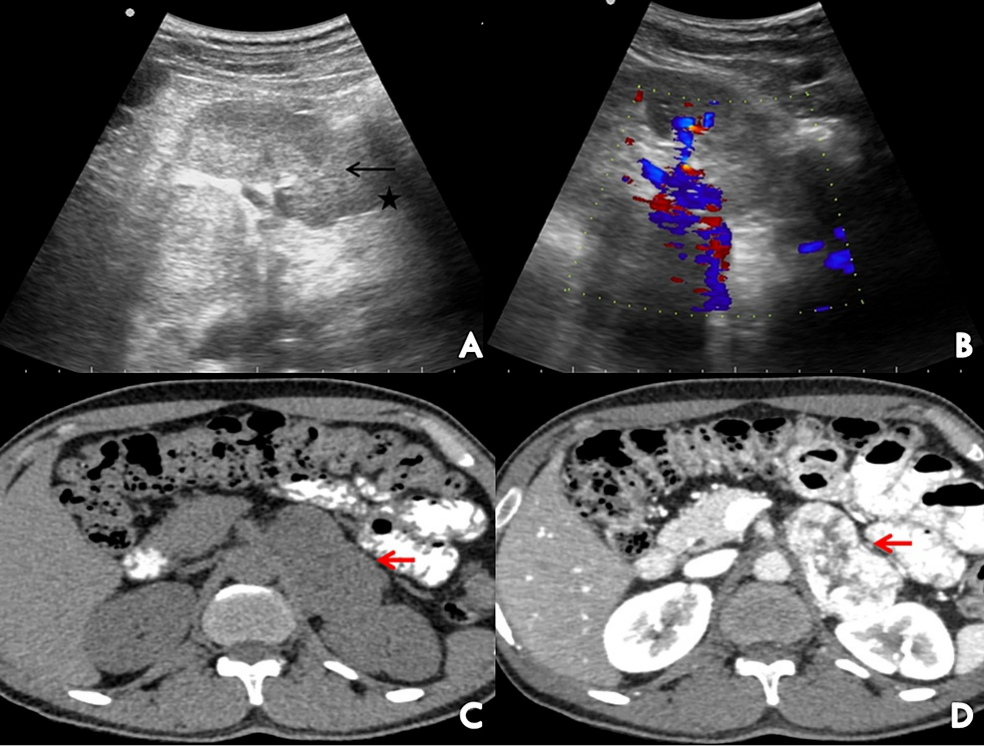
Ultrasound showed an oval heterogenous mass lesion (black arrow) seen superior to the left kidney (asterisk) (A) with prominent internal vascularity on color Doppler (B). Plain (C) and contrast-enhanced (D) axial images of the abdomen showed a well-defined hypodense solid lesion (red arrows) in the left adrenal gland with avid post-contrast enhancement.

**Figure 2:**
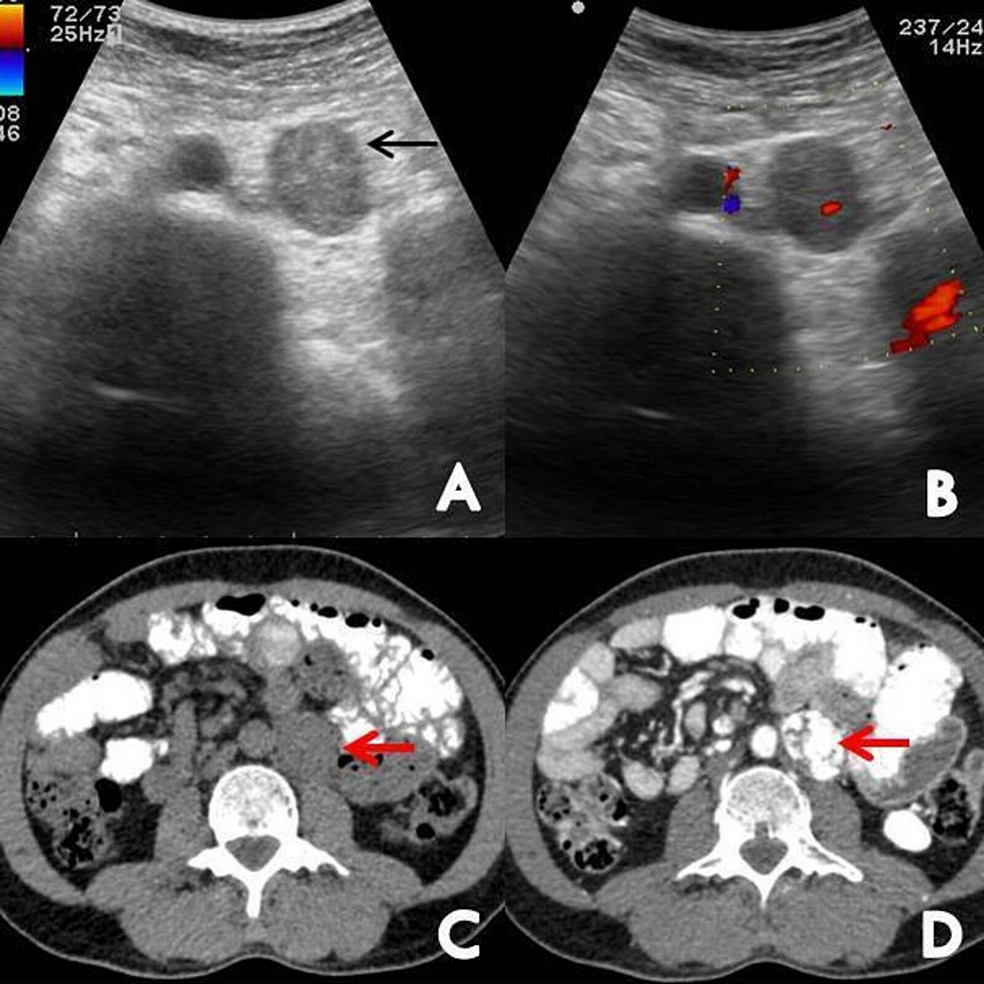
Ultrasound showed an oval heterogenous mass lesion seen in the left para-aortic region (black arrow) (A) with prominent internal vascularity on color Doppler (B). Plain (C) and contrast-enhanced (D) axial images of the abdomen showed a well-defined hypodense solid lesion in the left para-aortic region (red arrows) with avid post-contrast enhancement.

Transabdominal ultrasonography revealed two oval heterogeneously hypoechoic mass lesions showing internal vascularity on color Doppler, seen in the left suprarenal region displacing the splenic vessels and tail of the pancreas (Figure 1) and in the left lower para-spinal region (Figure 2).

Contrast-enhanced CT of the abdomen revealed a well-defined avidly enhancing solid lesion arising from the left adrenal gland at T12-L1 level measuring 64 x 45 x 52 mm [AP x TR x CC (anteroposterior x transverse x craniocaudal)] abutting the upper half of anterior surface of left kidney, left crus of the diaphragm and displacing the body and tail of pancreas superiorly (Figure 1). Another similar avidly enhancing solid lesion was seen in the left paraaortic region at L3 vertebral level measuring 41 x 28 x 29 mm [CC x TR x AP (craniocaudal x transverse x anteroposterior)] abutting the left lateral wall of the abdominal aorta (Figure 2). Both lesions showed absolute contrast washout of more than 60% and relative washout of more than 40%. These findings were highly suggestive of left adrenal pheochromocytoma and left para-aortic paraganglioma.

**Figure 3:**
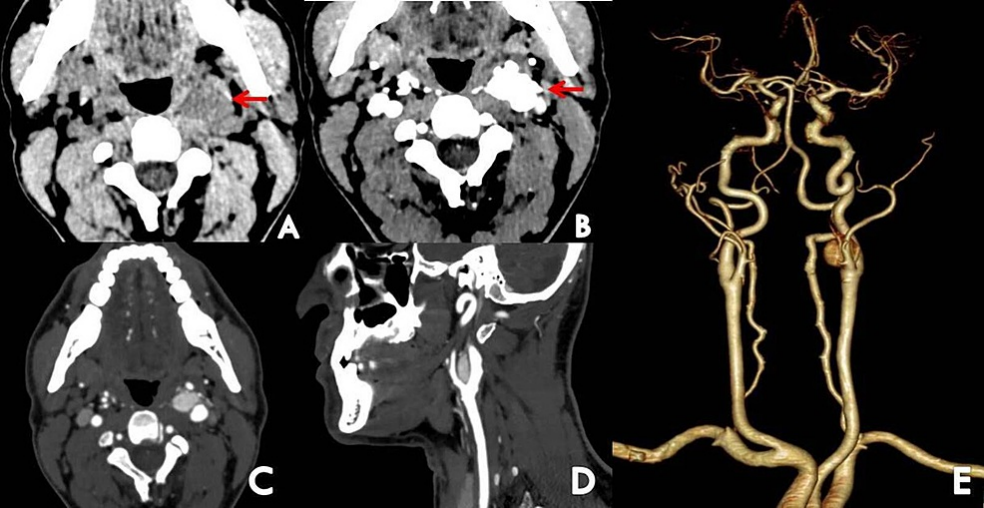
Plain (A) and contrast-enhanced (B) axial images of the neck and contrast-enhanced axial (C) and sagittal (D) CT reconstruction demonstrates a well-defined isodense mass lesion at the bifurcation of the left common carotid artery showing intense homogeneous post contrast enhancement (red arrows). 3D Coronal reformation from CT angiogram (E) showed the lesion causing splaying of the internal and external carotid arteries.

Contrast-enhanced CT of the neck was also done to screen for other sites of paragangliomas and revealed a well-defined isodense mass lesion measuring 17 x 15 x 11 mm (CC x TR x AP) at the division of the left common carotid artery splaying the external and internal carotid arteries and showing intense homogeneous post-contrast enhancement, suggestive of carotid body paraganglioma (Figure 3).

Screening test for secondary hypertension showed an abnormal increase of plasma-free metanephrine 4663 pg/ml (normal values < 57 pg/ml) and 24-hour urinary metanephrine of 420 μg/24 h (normal values 4-350 μg/24 h).

**Figure 4:**
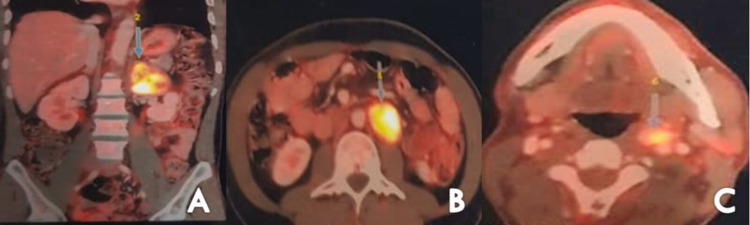
Fluorodeoxyglucose (18F-FDG) positron emission tomography revealed hypermetabolic soft tissue mass lesion in left adrenal gland (A), left paraaortic region (B), and in left carotid sheath at level of common carotid artery bifurcation (C).

18F-FDG (Fluorodeoxyglucose) positron emission tomography showed hypermetabolic soft tissue mass in the left suprarenal region, left para-aortic region and in the left carotid sheath at the level of common carotid artery bifurcation suggestive of multiple paragangliomas (Figure 4). No evidence of active disease elsewhere was seen.

**Figure 5:**
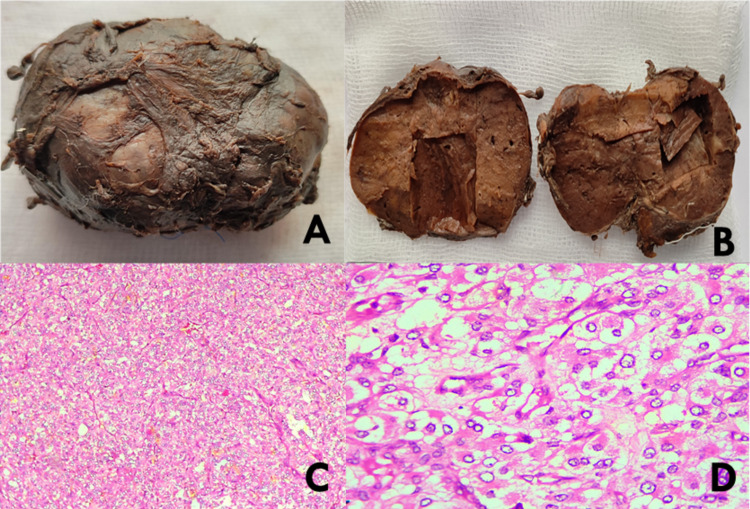
(A) Gross specimen of resected left adrenal mass. The tumor was well encapsulated. (B) Gross specimen of bisected left adrenal mass showed a soft and dark-reddish surface. (C) Haematoxylin and Eosin-stained section, x100 showing tumor composed of typical trabecular and nested pattern (Zellballen Pattern). (D) Haematoxylin and Eosin-stained section, x400 showing individual polygonal tumor cells with granular cytoplasm and round to oval clear nuclei.

**Figure 6:**
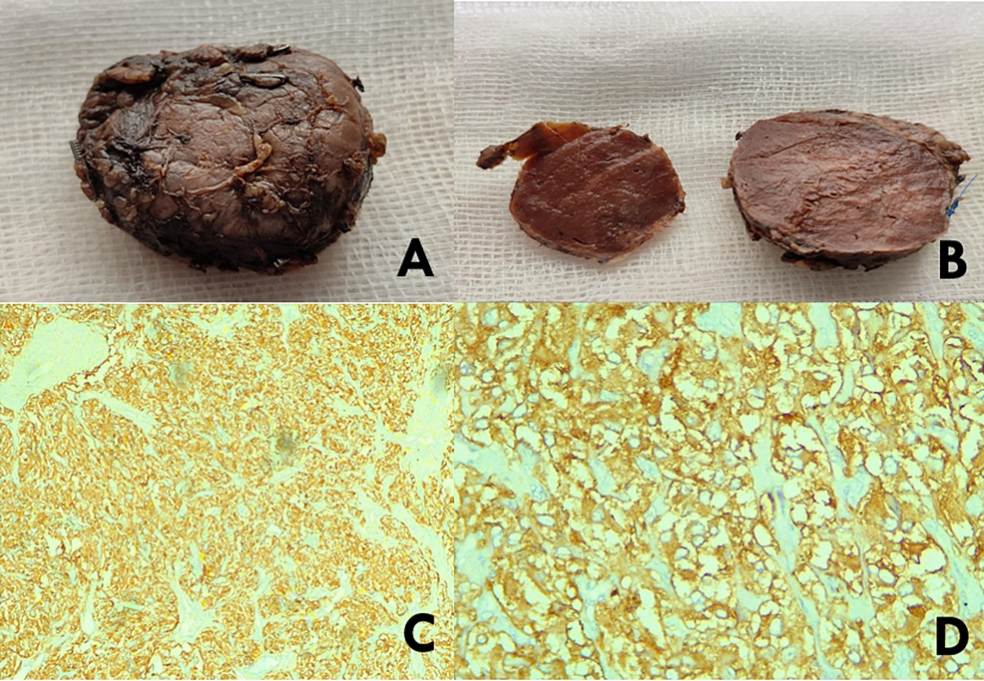
Gross image of resected left para-aortic mass. The tumor was well encapsulated (A). Gross specimen of bisected left para-aortic mass showed a soft and dark-reddish surface (B). Immunohistochemical examinations showed that it was strongly positive for chromogranin A (C) and synaptophysin (D) and confirmed neuroendocrine origin, supporting the diagnosis of paraganglioma.

After adequate control of hypertension by medical management, two of the three solid encapsulated tumors in the adrenal and left para-aortic region were resected. The resected tumors were soft, dark-reddish, and encapsulated. Histological examination revealed neoplasm consisting of groups of tumor cells giving the characteristic Zellballen pattern (Figure 5). Immunohistochemical staining was positive for chromogranin A and synaptophysin (Figure 6), hence confirming its neuroendocrine origin, supporting the diagnosis of paraganglioma.

After surgery, the patient’s blood pressure was within normal range without medications and plasma level of metanephrine decreased to 21 pg/ml. The patient was discharged with the plan for surgical resection of the carotid body paraganglioma in near future.

## Discussion

Paragangliomas commonly develop as solitary tumors. Multiple paragangliomas are rare and when present is usually more common in familial variety and associated with inherited syndromes such as neurofibromatosis, von Hippel-Lindau, paraganglioma syndrome, and multiple endocrine neoplasias. The number of multiple PGLs greatly increases in familial tumors and with germline mutations [6]. Multiple paragangliomas are uncommon in sporadic cases. Only 1% of sporadic cases present with multiple PGLs as compared to 20 to 80% in familial type [7].

Paragangliomas occur more frequently in the age group of 40-50 years. More than half of these tumors are functional, and patients frequently have symptoms due to excessive catecholamine output, including palpitations, headaches, sweating, and hypertension. Ten percent of paragangliomas can be occult and are detected incidentally at imaging study. Paragangliomas show intense post-contrast enhancement due to hypervascularity on CT. Smaller tumors are more likely to be homogeneous, unlike bigger ones which are inhomogeneous due to the areas of necrosis or hemorrhage within. Along with imaging, biochemical tests aid in diagnosis. Functional imaging with I-123 metaiodobenzylguanidine (a catecholamine analog) is used to confirm the diagnosis, identify other locations, and look for metastasis. Although most paragangliomas are benign and have a fair prognosis, they can be locally invasive or metastatic with either lymphatic or hematogenous dissemination to regional lymph nodes, lung and liver [4].

Due to the high vascularity of paragangliomas, biopsy should be avoided. Surgical resection is the best treatment and gives a good prognosis. Even though the surgical approach for paragangliomas is favourable, post-operative follow-up on a long-term basis using CT or MRI is recommended [8].

## Conclusions

Multiple paragangliomas in abdominal and neck origin without familial inheritance are exceedingly rare with limited cases reported. The possibility of paragangliomas should always be considered when hypervascular masses are encountered in certain paraganglia locations of the body. Presence of such a lesion must prompt further imaging of the common sites of paragangliomas for the detection of occult synchronous paragangliomas as was in our case. Furthermore, routine screening at timely intervals in patients previously diagnosed with paraganglioma may aid in the earlier detection of metachronous tumors. In our case, multiple paragangliomas were present in varied locations highlighting the need for screening of other sites of paragangliomas.
